# Delayed expression of cell cycle proteins contributes to astroglial scar formation and chronic inflammation after rat spinal cord contusion

**DOI:** 10.1186/1742-2094-9-169

**Published:** 2012-07-11

**Authors:** Junfang Wu, Ahdeah Pajoohesh-Ganji, Bogdan A Stoica, Michael Dinizo, Kelsey Guanciale, Alan I Faden

**Affiliations:** 1 Department of Anesthesiology & Center for Shock, Trauma and Anesthesiology Research (STAR), University of Maryland, School of Medicine, Baltimore, MD, 21201, USA; 2 Department of Neuroscience, George Washington University Medical School, Washington, DC, 20037, USA

**Keywords:** Contusive spinal cord injury, Cell cycle pathway, Cyclin-dependent kinases, Chronic, CR8, Astrogliosis, Inflammation, Rat

## Abstract

**Background:**

Traumatic spinal cord injury (SCI) induces secondary tissue damage that is associated with astrogliosis and inflammation. We previously reported that acute upregulation of a cluster of cell-cycle-related genes contributes to post-mitotic cell death and secondary damage after SCI. However, it remains unclear whether cell cycle activation continues more chronically and contributes to more delayed glial change. Here we examined expression of cell cycle-related proteins up to 4 months following SCI, as well as the effects of the selective cyclin-dependent kinase (CDKs) inhibitor CR8, on astrogliosis and microglial activation in a rat SCI contusion model.

**Methods:**

Adult male rats were subjected to moderate spinal cord contusion injury at T8 using a well-characterized weight-drop model. Tissue from the lesion epicenter was obtained 4 weeks or 4 months post-injury, and processed for protein expression and lesion volume. Functional recovery was assessed over the 4 months after injury.

**Results:**

Immunoblot analysis demonstrated a marked continued upregulation of cell cycle-related proteins − including cyclin D1 and E, CDK4, E2F5 and PCNA − for 4 months post-injury that were highly expressed by GFAP^+^ astrocytes and microglia, and co-localized with inflammatory-related proteins. CR8 administrated systemically 3 h post-injury and continued for 7 days limited the sustained elevation of cell cycle proteins and immunoreactivity of GFAP, Iba-1 and p22^PHOX^ − a key component of NADPH oxidase − up to 4 months after SCI. CR8 treatment significantly reduced lesion volume, which typically progressed in untreated animals between 1 and 4 months after trauma. Functional recovery was also significantly improved by CR8 treatment after SCI from week 2 through week 16.

**Conclusions:**

These data demonstrate that cell cycle-related proteins are chronically upregulated after SCI and may contribute to astroglial scar formation, chronic inflammation and further tissue loss.

## Background

Spinal cord injury (SCI)-induced astrogliosis and inflammation play a significant role in delayed secondary tissue damage that occurs for days, weeks and even months after the initial injury [1-8]. After SCI, astrocytes become hypertrophic, proliferate and show increased expression of GFAP. Hypertrophic astrocytes are the major cellular component of the glial scar, which is considered a physical and molecular barrier to CNS regeneration [5]. Reactive astrocytes produce several classes of growth-inhibitory molecules, including the family of extracellular matrix molecules known as chondroitin sulfate proteoglycans (CSPGs), which inhibit both *in vitro* and *in vivo* axonal regeneration [5,9,10]. Proliferation and activation of microglia, with resultant production of proinflammatory cytokines and neurotoxic molecules, are also implicated in secondary injury [3,11-15]. We have previously demonstrated that SCI in the rodent causes a delayed, sustained upregulation of proinflammatory genes such as C1qB, galectin-3, p22^PHOX^, gp91^PHOX^, CD53 and progranulin, among others [16,17]. p22^PHOX^ and gp91^PHOX^ are components of NADPH oxidase, which plays a key role in the production of reactive oxygen species [18-20]. The latter have cytotoxic effects, including induction of proinflammatory cytokine expression via MAPK and NFkB signaling [19-21]. Thus, modulation of reactive astrocytes and microglia represent important potential therapeutic targets for spinal cord injury.

We have shown that cell cycle-related genes and proteins are strongly upregulated immediately after SCI; they remain elevated for at least several weeks, and are associated with proliferation and activation of both astroglia and microglia [22-25]. Tian et al. also found that the upregulation of expression of cyclins A, B1, E and proliferating cell nuclear antigen (PCNA) appear as early as 1 day after injury and peak at day 3 following spinal cord hemisection [26]. However, it is not known if cell cycle activation continues more chronically following injury, resulting in persistent glial proliferation/activation that may contribute to late tissue loss.

It has been reported that CDK inhibitors can limit cell cycle activation and certain components of secondary tissue injury after neurotrauma [23,24,26-34]. We found that the non-selective CDK inhibitor flavopiridol reduced tissue damage and associated neurological dysfunction 1 month after impact SCI in rats [23,24]. However, because this drug inhibits most CDKs as well as transcription of cyclin D1, the role of specific CDKs after SCI has remained unclear. Olomoucine, a relatively selective CDK inhibitor, reduces neuronal apoptosis, suppresses astroglial scar formation and therefore ameliorates behavior outcome after spinal cord hemisection [26]. However, its potency for inhibition of purified CDKs and CDK activity in cell lines is relatively weak [35]. Recently, an N6-biaryl-substituted derivative of roscovitine, called CR8, was synthesized and optimized in an effort to generate second-generation roscovitine analogs with greater therapeutic potential compared to the parent compound [36].

In the present study, we evaluated the expression of cell cycle-related proteins up to 4 months after SCI. In addition, we examined a more clinically relevant delayed systemic treatment paradigm, using a newer and more potent roscovitine analog to assess the role of cell cycle activation in the progressive tissue loss and chronic astrogliosis after SCI.

## Methods

### Spinal cord injury

Contusive SCI was performed in adult male Sprague-Dawley rats weighing 275–325 g as previously described [24,37]. Briefly, rats were deeply anesthetized with sodium pentobarbital (65 mg/kg i.p.), and a moderate spinal cord contusion injury was induced at vertebral level T8 by dropping a 10-g weight from a height of 25 mm onto an impounder positioned on the exposed dura. Sham animals underwent the same procedure as injured rats, but received a laminectomy only, without weight drop. After injury, rats were placed into a heated cage to maintain normal core temperature until fully alert, and their bladders were manually expressed twice a day until a reliable bladder emptying reflex was established (10–14 days after SCI). The experimental protocols were approved by the University of Maryland School of Medicine Animal Care and Use Committee and met all NIH guidelines.

### Drug treatment

Following SCI, rats were randomly and blindly assigned to either drug or vehicle treatment group. Rats received intra-peritoneal (IP) injection of CR8 (second-generation roscovitine analog, 1 mg/kg, Tocris Bioscience) or an equal volume of vehicle once daily beginning 3 h post-injury and continuing for 7 days. CR8 was dissolved in sterile saline. This dose of CR8 was based on the results obtained from pilot studies *in vitro* and *in vivo*. More specifically, anti-apoptotic concentrations of CR8 in cultured cortical neurons were similar to those of flavopiridol, a potent pan-CDK inhibitor. Rats were sacrificed for histological/immunohistochemical study at 4 months post-injury.

### Immunoblot analysis

At 4 weeks and 4 months post-injury, rat spinal cord tissue (5 mm) centered on the injury site was collected and frozen on dry ice for Western analysis [38,39] with *n* = 4–5 rats per time point plus four laminectomy controls. Briefly, tissue was lysed in radioimmunoprecipitation assay (RIPA) buffer (Sigma) supplemented with 100 mM phenylmethylsulfonyl fluoride, 1× protease inhibitor cocktail, phosphatase inhibitor cocktail II and III (Sigma), then homogenized and sonicated. After centrifugation at 20,600 × g for 20 min, protein concentrations in supernatant were determined by the Pierce BCA method (Thermo Scientific). Normalized protein samples were denatured in LDS loading buffer. Each sample was from a different subject and run in an individual lane on 4 to 12% NuPAGE Novex Bis-Tris gradient gels (Invitrogen), and then transferred to nitrocellulose membranes (Invitrogen). After blocking in 5% nonfat milk for 1 h at room temperature, membranes were probed with antibodies against CDK4 (polyclonal, 1:1,000, Santa Cruz Biotechnology), cyclin D1 (polyclonal, 1:500, Neomarker), cyclin E (monoclonal, 1:500, Santa Cruz Biotechnology), E2F5 (polyclonal, 1:500, Santa Cruz Biotechnology), PCNA (polyclonal, 1:500, Santa Cruz Biotechnology), ionized calcium-binding adaptor molecule 1 (Iba-1,polyclonal, 1:1,000, Wako Chemicals) and p22^phox^ (polyclonal, 1:500, Santa Cruz Biotechnology) overnight at 4 °C followed by horseradish peroxidase-conjugated secondary antibodies (GE Healthcare) for 1 h at room temperature. The immunocomplexes were then visualized using SuperSignal West Dura Extended Duration Substrate (Thermo Scientific) and quantified by band densitometry of scanned films using the Gel-Pro Analyzer program (Media Cybernetics, Inc.) in the linear detection range. GAPDH was used as control for gel loading and protein transfer. Each sample was repeatedly run three times using the same blot and a pooled average was taken. The error bars in the Western blot quantification reflect variance across subjects and repeated runs of the same blots.

### Tissue processing and histopathology

At 4 weeks and 4 months after injury, rats were anesthetized and intracardially perfused with 200 ml of saline followed by 300 ml of 10% buffered formalin. The dissected spinal cords were post-fixed for 2 h and cryoprotected through a sucrose gradient. A 1.5-cm segment of spinal cord centered at the injury area was sectioned at 20-μm thickness and thaw-mounted onto Superfrost Plus slides (Fisher Scientific) by placing them serially on sequential sets of ten slides, each set representing a 200-μm length of spinal cord. A representative slide from each set was stained with eriochrome cyanine (ECRC) for myelinated white matter (blue). The lesion epicenter was identified as the section with the least amount of spared white matter [40].

### Estimation of lesion volume

Lesion volume was assessed using the Stereologer 2000 software (Systems Planning and Analysis, Alexandria, VA). Sections spaced 1 mm apart from 5 mm caudal to 5 mm rostral from the injury epicenter were stained with GFAP and DAB as the chromogen for lesion volume assessment based on the Cavalieri stereology method with a grid spacing of 200 μm. Lesion volume where GFAP is absent is expressed as a percentage of total volume including both areas of GFAP present and absent [24].

### Immunohistochemistry

Immunohistochemistry was performed on spinal cord coronal sections at specified distances rostral and caudal to the injury epicenter. Standard fluorescent immunocytochemistry on serial, 20-um-thick sections was performed as described previously [38]. The following primary antibodies were used: rabbit anti-CDK4 (1:100, Santa Cruz Biotechnology), rabbit anti-cyclin D1 (1:50, Neomarker), mouse anti-cyclin E (1:100, Santa Cruz Biotechnology), rabbit anti-E2F5 (1:100, Santa Cruz Biotechnology), rabbit anti-PCNA (1:100, Santa Cruz Biotechnology), rabbit or mouse anti-GFAP (1:500, Chemicon), rabbit anti-Iba-1 (1:500, Wako Chemicals) and rabbit anti-p22^phox^ (1:100, Santa Cruz Biotechnology). Fluorescent-conjugated secondary antibodies (Alexa 488-conjugated goat anti-mouse or rabbit, 1:400, Molecular Probes) were incubated with tissue sections for 1 h at room temperature. Cell nuclei were labeled with bis-benzimide solution (Hoechst 33258 dye, 5 ug/ml in PBS, Sigma). Finally, slides were washed and mounted with an anti-fading medium (Invitrogen). Immunofluorescence was visualized by tile scan using a Leica TCS SP5 II Tunable Spectral Confocal microscope (Leica Microsystems Inc., Bannockburn, IL). The images were processed using Adobe Photoshop 7.0 software (Adobe Systems, San Jose, CA). All immunohistological staining experiments were carried out with appropriate positive control tissue as well as primary/secondary-only negative controls.

### Function assessment

Rat hind limb locomotor recovery was assessed at 1 day post injury and weekly thereafter for up to 16 weeks using the Basso, Beattie and Bresnahan (BBB) open field expanded locomotor score [41]. In the BBB test, normal animals are given a score of 21, while animals with no hind-limb function are given a 0, with any combination of indicators of paralysis or regained function yielding scores in between. Rats were also scored on a battery of tests to determine recovery of hind limb motor and sensory function including: open field locomotion (motor score); withdrawal reflex to hind limb extension, pain and pressure; foot placing, toe spread and righting reflexes; maintenance of position on an inclined plane and swimming tests. Results of these tests are reported as a Combined Behavioral Score (CBS) [42]. Rats with normal function receive a score of 0, while rats with abnormal scores on all tests receive a score of 100. All rats were tested without knowledge of treatment group.

### Sampling and statistical analysis

All data are plotted as mean ± SEM where “*n*” is the number of individual animals. Western blot, lesion volume and stereological analyses were performed by an investigator blinded to treatment group. The expressions of various proteins (% of sham) were analyzed by using Kruskal-Wallis one-way ANOVA based on ranks, followed by Dunnett’s or Tukey’s post-hoc test (Sigma Stat Program, Version 3.5, Systat Software). BBB and CBS scores were analyzed with two-way ANOVA and repeated measures. All other statistical tests were performed using the GraphPad Prism Program, version 3.02 for Windows (GraphPad Software). A *p* < 0.05 was considered statistically significant**.**

## Results

### Spinal cord injury induces long-term changes in expression of cell cycle-related proteins

**Figure 1:**
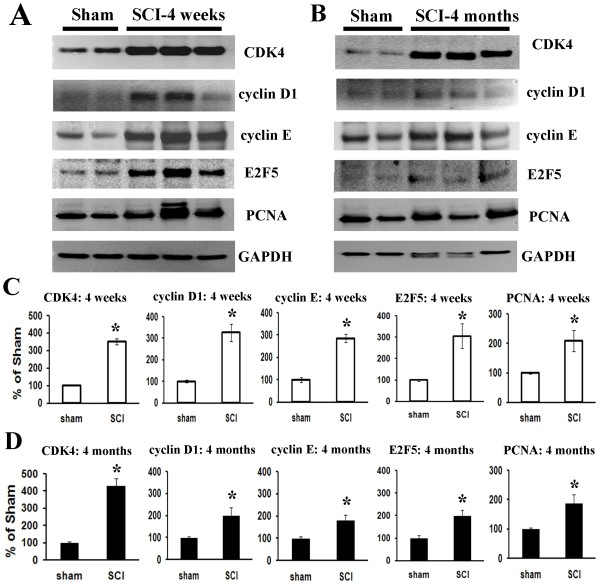
**Spinal cord injury induces long-term upregulation of expression of cell cycle-related proteins.** Analysis of expression of cell cycle proteins in intact and injured spinal cord at 4 weeks and 4 months post-injury was performed by Western blotting. (**A-B)** Representative immunoblots for cell cycle-related proteins (CDK4, cyclin D1 and E, E2F5 and PCNA) and the loading control (GAPDH). **(C-D)** Expression levels of cell cycle proteins were normalized by GAPDH, as estimated by optical density measurements, and expressed as a percentage of sham spinal cord. Quantitative analysis of Western blots showed significant upregulation of these cell cycle proteins at 4 weeks, which remained elevated for at least 4 months post injury. *p < 0.05 compared with the sham group. *n* = 4-6 rats per time point.

Quantitative analysis of Western blots showed that CDK4 expression was significantly increased at both 4 weeks (3.5 fold of sham) and 4 months (4.5 fold of sham) post SCI (*p* < 0.05, respectively Figure 1). The expressions of cyclin D1 and E were significantly increased at 4 weeks and 4 months post injury (Figure 1C and D). E2F5 expression levels were three fold that of sham at 4 weeks post injury and remained elevated (1.8 fold of sham) at 4 months. Levels of PCNA protein in spinal cord tissue were approximately two fold that of sham from 28 days through 4 months post-injury (Figure 1 A and B).

### Delayed expression of cell cycle proteins is associated with reactive astrocytes and chronic inflammation after SCI

**Figure 2:**
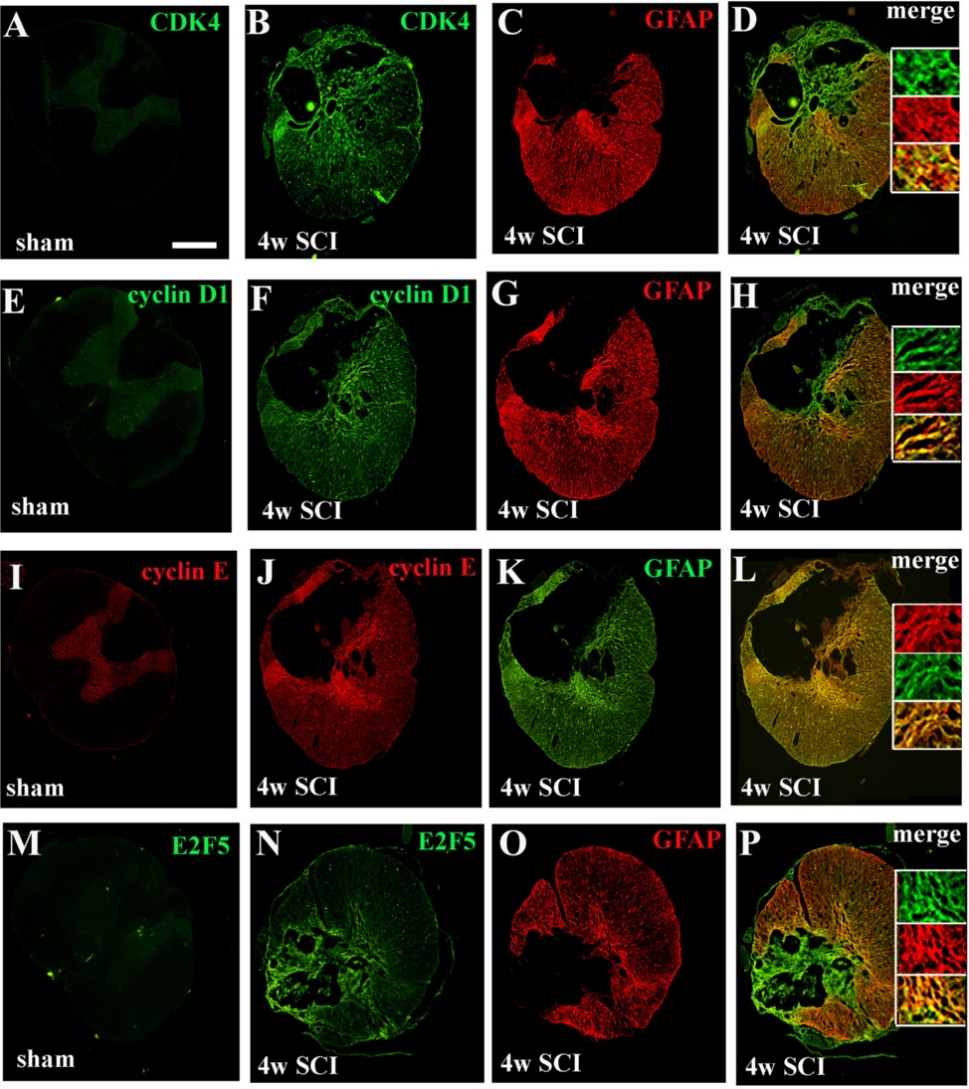
**Upregulated cell cycle proteins are associated with GFAP**^**+**^**reactive astrocytes at 4 weeks after SCI.** (**A-D**) Immunohistochemistry for qualitative assessment of CDK4 expression at 4 weeks after SCI. Coronal section in intact spinal cord showed that CDK4 was mostly expressed in the gray matter (**A**). CDK4 immunoreactivity was increased in the spared tissues surrounding the central lesion at 1 mm caudal to the epicenter (**B**), which co-labeled with GFAP^+^ astrocytes (C and insert in D). Note that CDK4^+^ cells also appeared in the central lesion area where GFAP^+^ astrocytes are absent. (**E-H**) Double-labeling immunohistochemistry revealed increased expression of cyclin D1 (**F**) and GFAP (**G**), and their co-localization (insert in H) in the injured tissue at 4 weeks post-injury. (**I-L**) Cyclin E^+^/GFAP^+^ cells were broadly observed in the spared tissue (insert in L). (**M-P**) E2F5 is not only expressed by GFAP^+^ hypertrophic astrocytes (insert in P) in the lesion scar border, but also in the central lesion area where GFAP^+^ astrocytes are absent. Scale bars = 500 μm.

**Figure 3:**
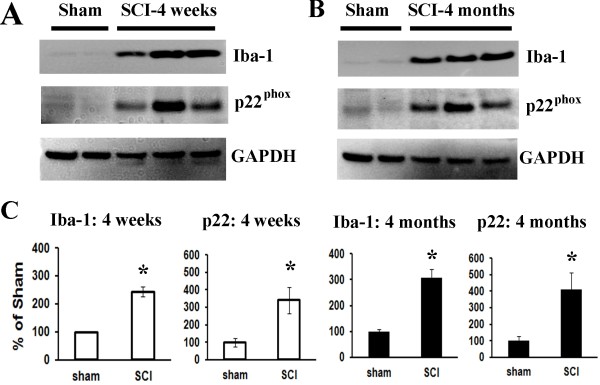
**Delayed upregulation of cell cycle proteins and GFAP is suppressed by cell cycle inhibition.** (**A**) Coronal section in intact spinal cord showed that cyclin D1 was mostly expressed in the gray matter (a). Cyclin D1 immunoreactivity at 4 months after injury was increased in the spared white matter surrounding the central lesion at 1 mm caudal to the epicenter (b), whereas this was attenuated by treatment with CR8 (c). GFAP immunoreactivity was weak in sham (d), but was strongly upregulated at 4 months post-injury (e). CR8 reduced expression of cyclin D1 (c) and GFAP (f), and their co-localization (i). (**B**) In the intact spinal cord, immunoreactivity of cyclin E (a), CDK4 (b) and E2F5 (c) was weakly detected in neurons across the gray matter. SCI resulted in increased expression of cyclin E (d), CDK4 (e) and E2F5 (f) at the site of the injury, which were attenuated in CR8-treated sections (g-i). Scale bars = 500 μm.

To determine the distribution and the cellular localization of increased cell cycle proteins in the injured rat spinal cord, we performed immunofluorescent double labeling of key cell cycle molecules and several cell-specific markers. In the intact spinal cord, immunoreactivity of CDK4, E2F5, cyclin D1 and E was weakly detected in neurons across the gray matter (Figure 2A, E, I and M). Four weeks after injury, expression was increased for each of these proteins in the spared tissues surrounding the central lesion at 1 mm rostral to the epicenter, especially in the border between spared tissue and the lesion (Figure 2 B, F, J and N). At 4 months post-injury, there was an increase in immunolabeling of cyclin D1 and E, E2F5 and CDK4 in contrast to sham tissue (Figure 3 Ab and Bd-f). Double-immunolabeling demonstrated that cyclin D1^+^/GFAP^+^ and cyclin E^+^/GFAP^+^ cells were readily apparent in the spared tissue (Figure 2H and L and Figure 3 Bb, e, h), whereas CDK4 and E2F5 were not only expressed by GFAP^+^ hypertrophic astrocytes in the lesion scar border, but also in the central lesion (Figure 2 C-D, G-H, K-L and O-P).

**Figure 4:**
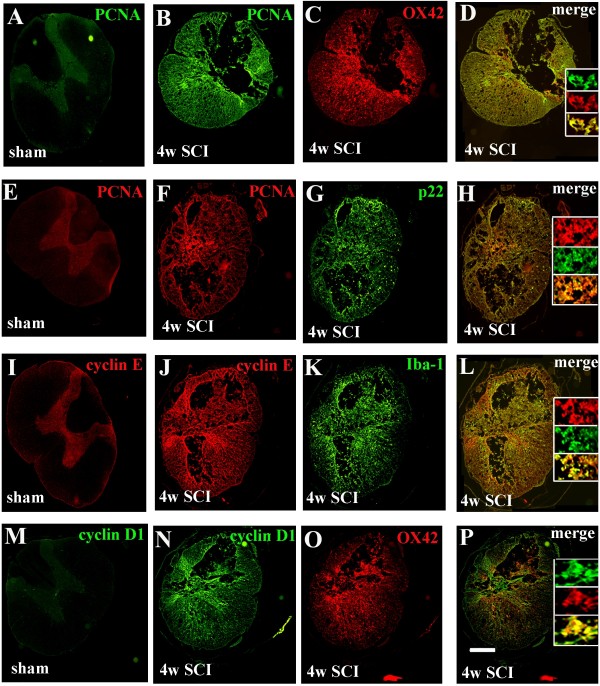
**Delayed inflammatory protein expression at 4 weeks and 4 months after contusive rat SCI.** Protein expression of microglia/macrophages marker Iba-1 and NADPH oxidase component p22^PHOX^ were analyzed using Western blotting at 4 weeks and 4 months post-injury. (**A-B**) Representative Western blots of Iba-1, p22^PHOX^ and the loading control at 4 weeks (A) and 4 months (B) after SCI. (**C**) Western blot analysis of Iba-1 and p22^PHOX^ protein expression indicated a significant increase at 4 weeks after SCI followed by a prolonged upregulation for up to 4 months post-injury. Bars represent mean ± SEM. *p < 0.05 compared with sham group. *n* = 4-6 rats per time point.

**Figure 5:**
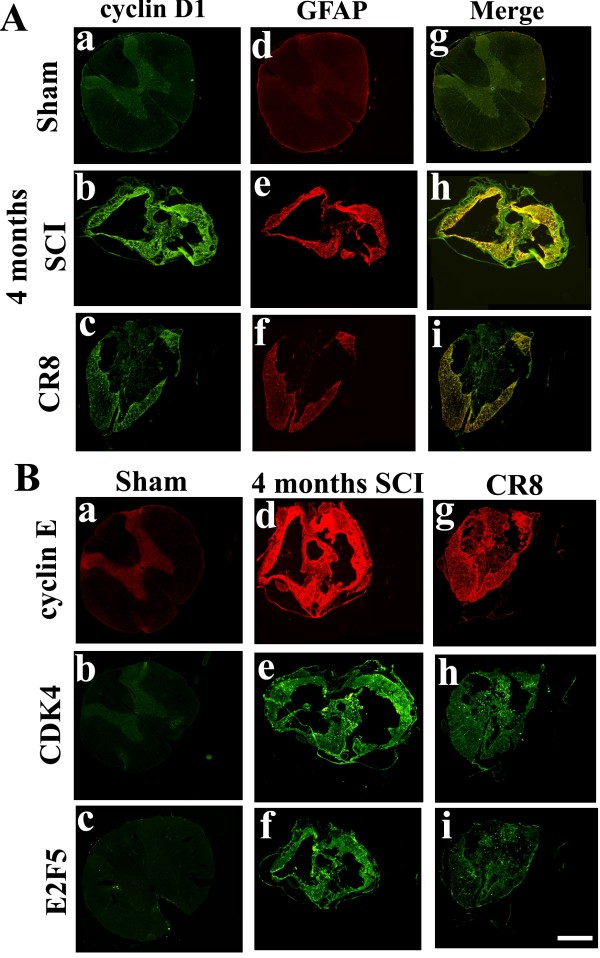
**The later upregulation of cell cycle proteins is associated with activated microglial/macrophages.** The expression of PCNA, cyclin D1 and E was evaluated by immunohistochemistry at 1.5 mm caudal to the epicenter of sham or injured spinal cord at 1 month post-injury. (**A-D**) PCNA expression was undetectable in sham, but was strongly upregulated in OX42^+^ microglia/macrophages. (**E-H**) The membrane bound component of the NADPH oxidase enzyme, p22, co-localized with PCNA^+^ microglia/macrophages. (**I-P**) Large numbers of cyclin E^+^ and D1^+^ cells in the injured coronal sections were co-labeled with Iba-1^+^ or OX42^+^ microglia/macrophages. Scale bars = 500 μm.

**Figure 6:**
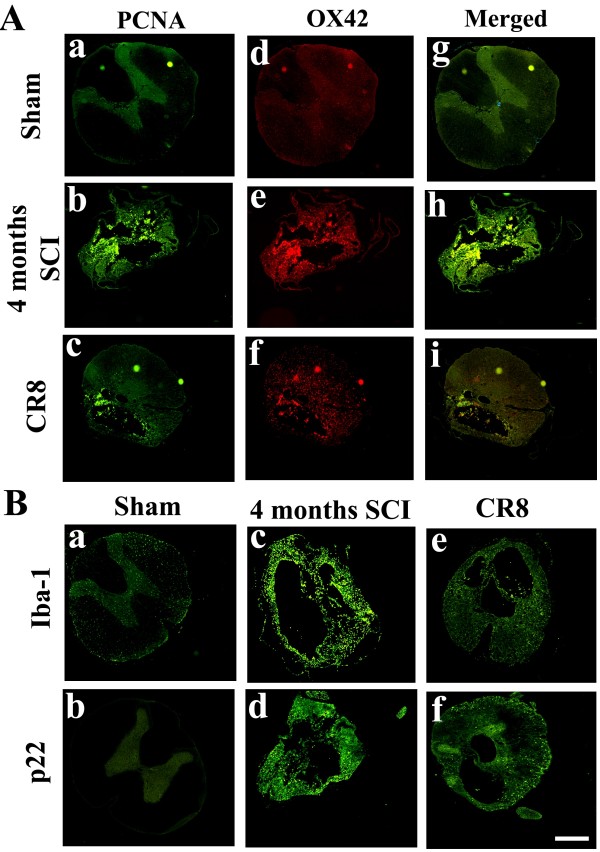
**Delayed systemic treatment with CR8 reduces chronic inflammatory protein expression at 4 months post-SCI.** (**A**) Double-labeling immunohistochemistry revealed increased expression of PCNA (b) and OX42 (e), and their co-localization (h) in the injured tissue at 4 months post-injury. Delayed systemic CR8 treatment reduced immunoreactivity in both PCNA and OX42 (c, f, i). (**B**) SCI induced the increase of expression of Iba-1 and p22^PHOX^ in contrast to sham tissue (c-d), whereas these increases were attenuated by CR8 treatment (e-f). Scale bars = 500 μm.

To examine whether increased expression of cell cycle-related proteins was associated with chronic inflammation, Western blotting and immunohistochemistry were performed for inflammatory markers Iba-1, p22^PHOX^ and CD11b (OX42) at 4 weeks and 4 months after SCI. Western blot analysis of Iba-1 protein expression indicated a 2.5-3.0-fold increase in injured spinal cord extracts compared to sham tissue (Figure 4 C). We also found a significant increase in p22^PHOX^ protein expression at 4 weeks (3.5 fold of sham) followed by a prolonged upregulation for up to 4 months post injury (Figure 4 A and B), consistent with our prior report [17]. Immunohistochemistry at 28 days and 4 months post-injury demonstrated an increase in immunolabeling of Iba-1, p22^PHOX^ and OX42 in contrast to sham tissue (Figure 5 C, G, K, O and Figure 6Ae and B c-d). Moreover, double-labeling immunohistochemistry revealed that large numbers of PCNA^+^, cyclin E^+^ and D1^+^ cells in the injured coronal sections were co-labeled with OX42, p22^PHOX^ or Iba-1 at 1.5 mm rostral to the epicenter (Figure 5 and Figure 6Ab, f, h). Overall, these data suggest that SCI-induced upregulation of cell cycle-related proteins occurs persistently for up to 4 months, and is associated with reactive astrocytes and activated microglia/macrophages.

### Delayed systemic cell cycle inhibition limits astrogliosis and delayed inflammatory protein expression

To further investigate the role of cell cycle pathway in SCI-induced astrogliosis and inflammation, injured rats were given the selective CDK inhibitor CR8 or vehicle by ip injection 3 h post-injury and daily thereafter for 7 days. Our pilot data showed that CR8 exhibits a 50-fold higher potency than roscovitine in *in vitro* neuroprotection. In the present study, rats were treated with 1 mg/kg CR8 or the equivalent volume of saline, and spinal cord sections were collected at 4 months after SCI for immunohistochemical study. GFAP is an indicator of astrocyte reactivity associated with glial scar formation [5]. Immunohistochemical analysis revealed increased expression of cyclin D1 (Figure 3Ab) and GFAP (Figure 3Ae), and their co-localization (Figure 3Ah) in the spared tissue surrounding the lesion site. In contrast, CR8-treated rats showed less immunoreactivity for both cyclin D1 and GFAP (Figure 3Ac, f, i). In addition, SCI resulted in increased expression of cyclin E, CDK4 and E2F5 at the site of the injury (Figure 3Bd-f), which were attenuated by CR8 treatment (Figure 3Bg-i).

Chronic inflammation was also evaluated by immunohistochemical analysis of spinal cord sections using Iba-1, p22^PHOX^ and OX42. Double-labeling immunohistochemistry revealed increased expression of PCNA (Figure 6Ab) and OX42 (Figure 6Ae), and their co-localization (Figure 6Ah) in the injured tissue at 4 months post-injury. CR8 treatment reduced immunoreactivity for both PCNA and OX42 (Figure 6Ac, f, i). SCI increased expression of Iba-1 and p22^PHOX^ as compared to sham (Figure 6Bc-d); these changes were attenuated by CR8 treatment (Figure 6Be-f). Taken together, these data demonstrated that delayed systemic treatment with a novel, selective and potent CDK inhibitor reduced astrogliosis and microglial proliferation at 4 months post-SCI.

### Cavity formation is progressive after chronic contusion and is attenuated by CDK inhibition

**Figure 7:**
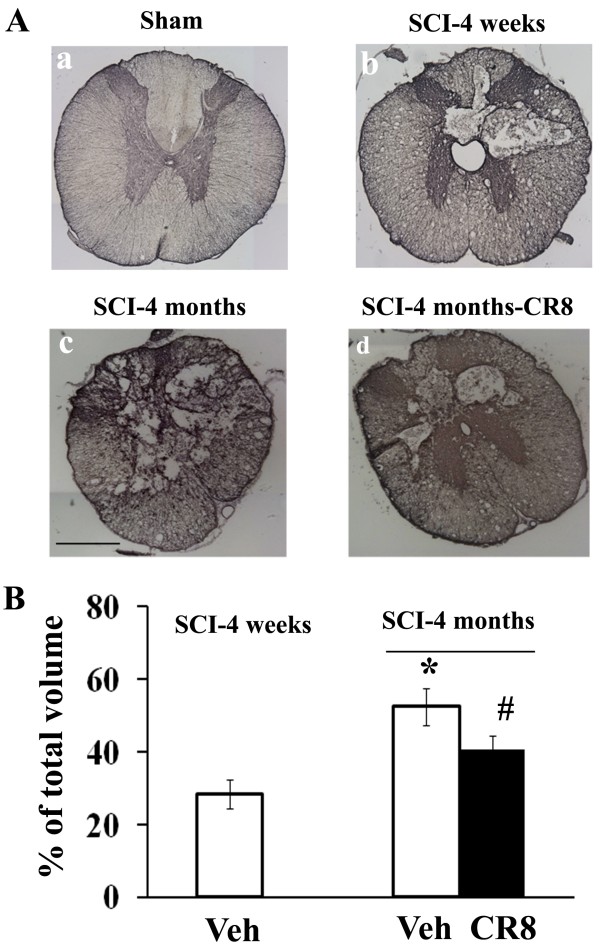
**Cavity formation is progressive after chronic contusion and is attenuated by CDKs inhibition.** SCI-induced lesion volume/cavity formation was measured with GFAP/DAB staining and analyzed by unbiased stereological techniques. (**A**) Representative images showed lesion cavity at 3 mm rostral to the injury center at 4 weeks (b) and 4 months (c) after SCI. Scale bars = 500 μm. (B) Histological assessment showed that 4 months-SCI rats developed a larger lesion cavity (52.4 ± 5.0% of total volume) than that in 4 weeks-SCI animals (28.4 ± 3.9% of total volume). Notably, a significant reduction in lesion volume was found in rats that received delayed systemic treatment of CR8 at 4 months post-injury (40.8 ± 3.6% of total volume). *p < 0.05 vs. SCI-4 weeks Veh group; ^#^*p* < 0.05 vs. SCI-4 months Veh group. *n* = 6 rats/group.

SCI-induced lesion volume/cavity formation was measured with GFAP/DAB staining at 1 and 4 months after SCI and analyzed by unbiased stereological techniques. Histological assessment showed that tissue collected 4 months post-SCI possessed a larger lesion cavity (52.4 ± 5.0% of total volume) than that at 1 month after injury (28.4 ± 3.9% of total volume), indicating progressive damage in the injured spinal cord (Figure 7). Notably, a significant reduction in lesion volume was found in CR8 treated rats at 4 months post-injury (40.8 ± 3.6% of total volume, Figure 7A and B). These reductions occurred in both white and gray matter, with an overall decrease in cavity formation and tissue loss.

### Cell cycle inhibition favors functional recovery after SCI

**Figure 8:**
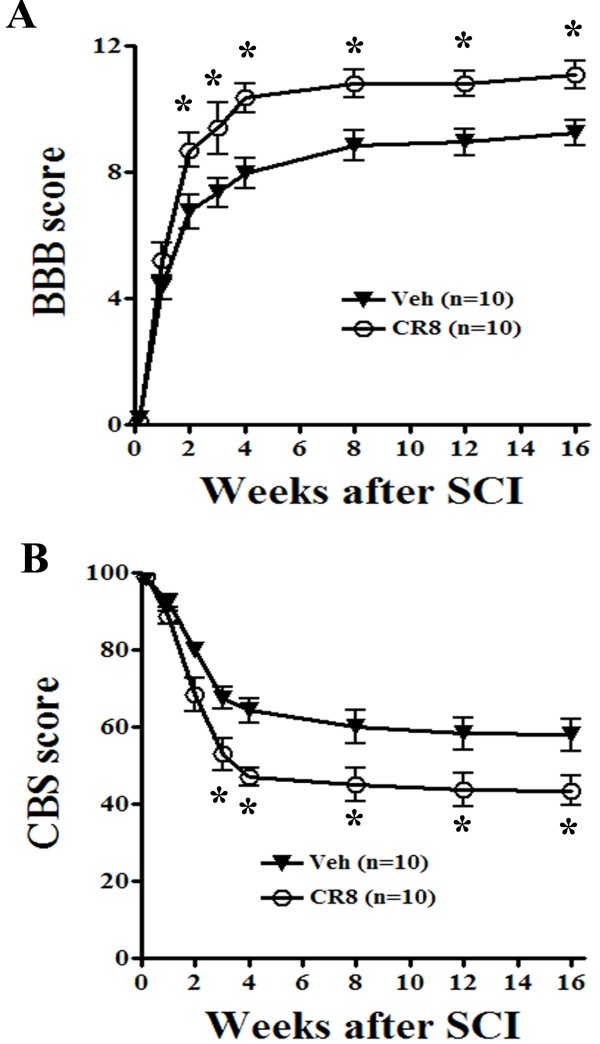
**Cell cycle inhibition by CR8 significantly improves functional recovery after SCI.** Both BBB and CBS scores were evaluated 1 day post injury and weekly thereafter in drug- vs. vehicle-treated rats. Both BBB and CBS tests showed a significant beneficial effect of delayed systemic CR8 treatment on functional recovery. The effect of treatment was statistically significant beginning at week 2 as measured by BBB score and beginning at week 3 as measured by CBS score. Both measures showed significantly improved recovery chronically at 4–16 weeks after SCI. *p < 0.05 vs. vehicle group at indicated time point.

To further address whether inhibition of cell cycle-related proteins improves neurological outcome, we next examined whether this same treatment strategy could influence long-term functional recovery after SCI. We assessed the behavior of CR8 and vehicle-treated rats over the 16 weeks after injury. Testing was performed 1 day after injury and weekly thereafter using the BBB test of hind limb locomotor function [41] and the Combined Behavioral Score (CBS), an evaluation of overall hind limb sensory-motor deficits [42]. Both BBB and CBS tests showed significantly improved functional recovery after delayed systemic CR8 treatment (Figure 8). The effect of treatment was statistically significant beginning at week 2 as measured by BBB score (Figure 8 A) and beginning at week 3 as measured by CBS score (Figure 8 B). Both measures showed significantly improved recovery chronically from 4–16 weeks after SCI.

## Discussion

Our current results demonstrate that SCI results in a marked, chronic upregulation of the expression of cell cycle-related proteins associated with reactive astrocytosis and microglial proliferation. Delayed systemic administration of CR8 limited chronic upregulation of cell cycle proteins and improved functional recovery up to 4 months post-SCI; this was associated with reduced astrogliosis and chronic inflammation that may contribute to the observed progressive tissue loss and glial scar formation.

SCI causes secondary biochemical changes that persist for months after SCI. The role of reactive astrocytes in the restorative stage after injury is complex, as they secrete numerous bioactive substances − including cytokines, antioxidants, recognition molecules and growth factors − that can be either neurotrophic or neurotoxic [43,44]. GFAP expression and immunoreactivity were increased at 1 month and 4 months after SCI. Several cell cycle proteins were upregulated in GFAP^+^ reactive astrocytes concentrated in the boundary zone between spared tissue and the lesion; treatment with a specific CDK inhibitor after SCI reduced the sustained upregulation of cell cycle protein expression as well as GFAP immunoreactivity. Taken together, our data demonstrate chronic cell cycle activation in reactive astrocytes after SCI, which may contribute to the glial scar formation. Thus, the ability of cell cycle inhibitors to limit scar formation may facilitate endogenous restorative potential.

Recent studies demonstrated a secondary peak of inflammation as late as 2 months post-injury [45,46]. We have shown that SCI in the rodent is followed by sustained upregulation of a cluster of proinflammatory genes for up to 6 months that may contribute to the continuation of damage in the injured cord [16,17]. Although microglia have both neurotoxic and neuroprotective effects [20,47,48], considerable experimental data suggest that post-traumatic inflammation, including microglial activation, contributes to chronic cell damage and progressive tissue loss [30,49,50]. Indeed, activated microglia and release of associated inflammatory factors has been indicated as an important contributing factor for many acute and chronic neurodegenerative disorders [51,52]. The present study confirms similar results evidenced by increased expression and immunoreactivity of the inflammatory markers, Iba-1, CD11b and a core component of the NADPH oxidase enzyme, p22^PHOX^; the increased cell cycle protein expression observed was co-expressed with these inflammatory markers in activated microglia as late as 4 months after SCI. In agreement with our previous findings [23,24,34], we detected reduction of inflammation in the SCI rats treated with a CDK inhibitor − including decreased immunoreactivity of Iba-1, CD11b and p22^PHOX^. Together, these data suggest that suppression of chronic inflammation by cell cycle inhibition may account, at least in part, for the progressive tissue loss after SCI. The results also suggest that persistent cell cycle activation after injury may reflect a positive feedback loop that can be interrupted with sub-acute cell cycle inhibitor administration.

Cell cycle proteins are also expressed in other cell types of the CNS [53,54], such as oligodendrocytes and infiltrating Schwann cells, which contribute to myelin repair in the injured spinal cord [55]. We recently reported increases in the myelinated white matter area and expression of myelin basic protein in flavopiridol-treated injured rats [24]. However, it remains unclear whether cell cycle inhibition increases remyelinated axons by oligodendrocytes and Schwann cells, or reduces chronic progressive demyelination. We showed CDK4 and E2F5 are highly expressed in the central lesion areas where astrocytes are absent but p75^+^ Schwann cells have infiltrated [24,38]. Postnatal Schwann cell proliferation has been known to be strictly and uniquely dependent on CDK4 [56]. However, further investigation is required to elucidate the mechanisms by which cell cycle inhibition modulates myelination after SCI.

CR8 exhibits a 50-fold higher potency than roscovitine in different cell lines, possibly owing its added efficacy to more potent inhibition of CDKs 1, 2, 5, 7 and 9, and increased solubility, cell permeability and enhanced intracellular stability [36,57]. More recently, we reported that CR8 at a single dose 20 times less than roscovitine [29,30] provides superior neuroprotection to the parent compound [58]**.** Given the increased potency and efficacy of CR8 as compared to earlier purine analog types of CDK inhibitors, this drug was used systemically in the present study. CR8 treatment limited sustained upregulation of cell cycle protein expression, as well as chronic reactive astrocytosis and microglial activation. Significantly reduced lesion volume and improved long-term functional recovery were also observed, suggesting that chronic cell cycle activation may contribute to secondary injury and expansion of the lesion site after SCI.

In summary, we provide evidence that SCI is accompanied by a prolonged, sustained upregulation of cell cycle-related protein expression that may contribute to the development of glial scar formation, chronic inflammation and progressive tissue loss. Blockade of cell cycle pathways by a CDK inhibitor significantly reduces delayed upregulation of cell cycle proteins, limits astrogliosis and chronic inflammation, and subsequent lesion progression, with marked improvement in functional recovery. Thus, sustained cell cycle dysregulation may contribute to the chronic progressive secondary injury after SCI.

## Abbreviations

CDKIs, Cyclin-dependent kinase inhibitors; CDKs, Cyclin-activated kinases; SCI, Spinal cord injury; BBB, Basso Beattie and Bresnahan locomotor rating scale; CBS, Combination behavioral scores; NADPH, Nicotinamide adenine dinucleotide phosphate; MAPK, Mitogen activated protein kinase; NADPH, Nicotinamide adenine dinucleotide phosphate; NFκB, Nuclear factor κ B; PCNA, Proliferating cell nuclear antigen; E2F5, E2 promoter binding factor 5; CSPG, Chondroitin sulfate proteoglycans; Iba-1, Ionized calcium binding adaptor molecule 1.

## Competing interests

The authors declare that they have no competing interests.

## Authors’ contributions

JW conceived the study and carried out the SCI surgeries, rat behavior study, immunoblotting and wrote the manuscript. APG participated in the SCI surgeries. BAS and MD carried out the immunohistochemistry and stereology studies. KG carried out the immunoblotting studies. AIF participated in the design of the study and wrote the manuscript. All authors read and approved the final manuscript.
